# Progress in exosome associated tumor markers and their detection methods

**DOI:** 10.1186/s43556-020-00002-3

**Published:** 2020-08-14

**Authors:** Mengjiao Shen, Kaili Di, Hongzhang He, Yanyan Xia, Hui Xie, Rongrong Huang, Chang Liu, Mo Yang, Siyang Zheng, Nongyue He, Zhiyang Li

**Affiliations:** 1 Department of Clinical Laboratory, the Affiliated Drum Tower Hospital of Nanjing University Medical School, Nanjing, 210008 China; 2 Shanghai Health Development Research Center, Shanghai, China; 3 Captis Diagnostics Inc, Pittsburgh, PA 15213 USA; 4 Department of Biomedical Engineering, the Hong Kong Polytechnic University, Hunghom, Kowloon, Hong Kong, People’s Republic of China; 5 Department of Biomedical Engineering and Electrical & Computer Engineering, Carnegie Mellon University, 5000 Forbes Avenue, Scott Hall 4N211, Pittsburgh, PA 15213 USA; 6 State Key Laboratory of Bioelectronics, School of Biological Science and Medical Engineering, Southeast University, Nanjing, 210096 China

## Abstract

Exosomes are secreted by cells and are widely present in body fluids. Exosomes contain various molecular constituents of their cells of origin such as proteins, mRNA, miRNAs, DNA, lipid and glycans which are very similar as the content in tumor cells. These contents play an important role in various stages of tumor development, and make the tumor-derived exosome as a hot and emerging biomarker for various cancers diagnosis and management in non-invasive manner. The present problems of exosome isolation and detection hinder the application of exosomes. With the development of exosome isolation and detection technology, the contents of exosomes can be exploited for early cancer diagnosis. This review summarizes the recent progress on exosome-associated tumor biomarkers and some new technologies for exosome isolation and detection. Furthermore, we have also discussed the future development direction in exosome analysis methods.

## Development on exosome tumor markers

Extracellular vesicle (EV) includes exosomes, microvesicles and apoptotic bodies. These vesicles have different size and biogenesis. Exosomes are complex 20–100 nm vesicles and generate in a way that intracellular multivesicular bodies (MVBs) containing intraluminal vesicles (ILVs) fuse with the plasma membrane [1]. Larger vesicles, microvesicles (100 nm–1 μm) and apoptotic bodies (1–5 μm), are released directly from the budding and fission of the plasma membrane [2]. In the past decades, researchers have become increasingly interested in the role of EVs, especially exosomes, in diseases.

Exosomes contain various molecular constituents of their cell of origin such as proteins, RNAs, DNA, lipid glycans. Therefore, tumor-derived exosomes could tell the physiological and pathological states of parent tumor cells, and emerged to be a hot cancer biomarker in liquid biopsy field [3]. Given the rich molecular composition of exosomes and easy availability of liquid biopsy sample, many researchers [4] are pursuing to develop non-invasive diagnostic methods with higher sensitivity and specificity based on exosome, which has very high potential to help early diagnosis, treatment evaluation, and prognostic analysis of the disease. In this section, we have summarized the application of exosomes in tumor diagnosis based on its amount and molecular compositions.

### Level of exosomes in tumor diagnosis

Studies show that the level of exosomes in plasma was significantly higher in cancers (such as ovarian cancer [5] and non-small-cell-lung cancer [6]) patients than that of healthy controls [7]. Therefore, many researchers hypothesize that levels of exosome in bodily fluid can serve as a potential diagnostic biomarker in cancer patients. Logozzi et al. [8] investigated the amount of tumor-derived exosome in mouse cancer model, and it was found that the levels of exosomes was correlated with tumor size. In another study, Liu Q et al. [9] found that level of exosome in plasma increases with tumor stage progression in 208 non-small cell lung cancer (NSCLC) cohort patients (*P* < 0.001). Furthermore, Yasunori et al. [10] isolated and quantified exosomes from plasma in esophageal cancer patients (*n* = 66), and revealed that higher level of exosome was obtained in malignant patient than that of non-malignant patients (*n* = 20) (*P* = 0.0002). Additionally, both of Liu et al. [9] and Taylor et al. [5] found that the level of exosome in plasma could be a prognostic biomarker in non-small-cell lung cancer and ovarian cancer, in which higher level of exosome is an indicator of poor prognosis. With the interesting finding from those clinical studies, the states of cancer development can be predicted by analyzing the levels of exosomes in biofluid samples. However, the sensitivity of analyzing cancer and cancer staging was highly negated by the high background signal from high level of normal cell-derived exosomes. Therefore, it is very hard to make a cut-off line in cancer diagnosis if we count the level of total exosome in plasma. However, the sensitivity and specificity of cancer diagnosis should be significantly enhanced if tumor-derived exosome could be selectively isolated or enriched from bodily fluid.

### Exosome proteins in tumor diagnosis

Exosome cargos contain rich information of proteins, such as skeletal protein, secretory associated protein etc. Interestingly, tumor-derived exosomes also contain proteins from their mother cells, making them an attractive biomarker for cancer diagnosis. Extensive studies found that exosome surface protein, intrinsic protein, and protein modification are significant biomarkers with potential clinical applications in cancer diagnosis. Table 1 summarizes the newly discovered protein biomarkers in tumor-derived exosome in recent years.

**Table 1:** Protein markers in exosome-based tumor diagnosis

Tumor category	Protein markers in exosome	Change in tumorigenesis
colorectal cancer	Copine III [11]	up-regulation
	CD147 [12]	up-regulation
pancreatic ductal adenocarcinoma	GPC-1 [13, 14]	up-regulation
Gastric cancer	HER-2/neu, EMMPRIN, MAGE-1, C-MET [15]	up-regulation
	TRIM3 [16]	down-regulation
Prostate cancer	PSA [17]	up-regulation
	ephrinA2 [18]	up-regulation
	survivin [19]	up-regulation
melanoma	(phospho)Met [20]	up-regulation
	caveolin-1 [21]	up-regulation
Renal cell carcinoma (RCC)	MMP-9, DKP4, EMMPRIN, PODXL [22]	Expression alone in the tumor derived exosomes
non-small-cell lung carcinoma	EGFR, KRAS, claudins and RAB-family proteins [23]	up-regulation
	CD151, CD171 and tetraspanin 8 [24]	up-regulation

#### Protein expression level

With rapid development of mass spectrometry and other protein identification technologies, many differentially expressed proteins in tumor cells have been discovered. Sandfeld-Paulsen et al. [25] found that CD151, CD171, and tetraspanin 8 are biomarkers for lung cancer diagnosis, those proteins were found to be powerful to distinguish cancer patients from healthy control. In other studies, exosomes were found to have great potential in breast cancer diagnosis. For example, the level of glypican-1 (GPC-1A) was found to be upregulated in 3/4 cancer patients [26]. Exosome protein survivin-2B was found to be a good biomarker in breast cancer diagnosis [27]. In one prostate cancer diagnosis study, it showed that levels of CLDN3 in exosome were higher in patients with Gleason≥8 tumors than that patients with benign prostatic hyperplasia (*p* = 0.012) and Gleason 6–7 tumors (*p* = 0.029), and higher levels of annexin (CD62, CD81), heat shock proteins (Hsp70, Hsp90) and many signal molecules (TGF-β2, TNF-α, IL-6 TSG101) were expressed in prostate cancer cell-derived exosome cultured in hypoxic condition than that of normally cultured cells. Additionally, Fu et al. [28] found that level of TRIM3 protein in serum exosomes decreased in gastric cancer patients. TRIM3 plays a role as tumor inhibition in gastric cancer, and TRIM3 knockdown can promote the growth and metastasis of gastric cancer by regulating stem cell factor and EMT regulator. By surveying the clinical studies on protein markers in exosome, most studies detected the levels of protein expression in total exosomes in bodily fluid. But they cannot avoid the interference from protein expressed in normal cell-derived exosomes, which decrease the sensitivity and specificity of protein biomarkers in cancer diagnosis. Therefore, technologies for tumor cell-derived subpopulation exosomes enrichment should be pursued as well to increase the sensitivity and specificity of cancer diagnosis.

#### Protein post-translational modification

Post-translational modification (PTM) is involved in protein sorting mechanism in exosome. The types of protein modifications in exosome include phosphorylation, ubiquitination, oxidation, myristoylation, GPI-anchor, citrullination, glycosylation, and SUMOylation [29]. Recent studies have shown the potential of protein modifications in exosome as a novel biomarker in diagnosis and prognosis of certain diseases. Since exosomes represent their original cancer cells, the level of their phosphorylation in EGFR can be a good biomarker in monitoring anti-tumor treatment effect [30]. Tao et al. [31] found that 144 of these phosphorylated protein levels in exosome were significantly elevated in cancer patients by comparing 30 breast cancer patients with 6 healthy control patients. Changes in glycosylation are very common in many types of tumor-derived exosomes. N- and O-glycosylated GPI-anchor CD24 in exosome is an established marker for poor prognosis in ovarian and other carcinomas [32, 33]. And bisecting GlcNAc-containing-glycans and high mannose glycans were found to be ovarian cancer biomarkers via glycomics analysis of EVs glycoproteins from ovarian cancer cells [34, 35]. Increased levels of glycosylation are often associated with changes in tumor aggressiveness. GlcNAcylation of many exosome proteins were found significantly increased in EVs from metastatic colorectal cancer cells [36], and this phenomenon of highly glycosylated extracellular matrix metalloproteinase (EMMPRIN) was observed with increased concentration in metastatic breast cancer as well [37]. Therefore, protein modification in exosome provides a totally new path for cancer diagnosis. However, due to the tremendous challenge in PTM identification technology, clinical evidence of exosome protein PTM needs further investigation.

### Exosome nucleic acids in tumor diagnosis

In April 2019, the research team of Robert J. Coffey re-evaluated the contents of exosomes and concluded that small cell extracellular vesicles (sEVs) do not contain DNA. A possible explanation is that different methods of exosome extraction are used in different studies, which in turn leads to differences in the content of exosomes and the subgroup of exosomes. Too strict an exosome isolation strategy may result in the loss of DNA-containing vesicles, which are too low to be detected. Recently, many studies have shown that DNA is detected in exosomes. Akira Yokoi et al. showed that genomic DNA (gDNA) and nucleoprotein exist in exosomes, and revealed exosome DNA potential diagnosis biomarker of ovarian cancer [38].

Recent studies on extracellular RNA (exRNA) including miRNA, long non-coding RNA (lncRNA), circRNA and tRNA-derived small RNA (tsRNA) have highlighted the potential of these biomolecules and vehicles as molecular signatures of disease, especially on prominent paradigm shift in the field of oncology. Although the nature of those RNAs in exosomes is not quite clear, much effort has been devoted to investigate their clinical application in cancer diagnosis. For example, high level of miR-105 in exosome can be an indicator of tumor metastasis and disease diagnosis [39]. Scientists also found increased level of LISCH7 mRNA in plasma EVs from colon cancer patients [40]. The tsRNA content in exosome has also become an attractive nucleic acid marker in recent years. Lei Zhu et al. found a large number of tsRNAs in exosome and some tsRNAs were significantly increased in plasma exosomes of liver cancer patients [41]. The nucleic acid biomarkers in exosome for tumor diagnosis are summarized in Table 2.

**Table 2:** Nucleic acid biomarkers in exosome for tumor diagnosis

Tumor category	Nucleic acid markers in exosome	Change in tumorigenesis
Pheochromocytoma and paraganglioma.	dsDNA with RET, VHL, HIF2A, and SDHB mutations [42]	mutation
Pancreatic cancer	miR-1246, miR-4644, miR-3976 and miR-4306 [43]	up-regulation
	miR-17-5p and miR-21 [44]	up-regulation
	circ-IARS (RNA) [45]	up-regulation
Lung cancer	miR-378a, miR-379, miR-139-5p, and miR-200b-5p [46]	up-regulation
	let-7 g-5p, mir-24-3p, mir-223-3p [47]	up-regulation
	mir-7-5p, mir-424-5p [47]	up-regulation (exosome in bronchoalveolar lavage)
Primary central nervous system lymphoma	miR-21 [48]	up-regulation
Glioblastoma multiforme	RNU6–1 (noncoding RNA), miR-320, miR-574-3p [49]	up-regulation
Endometrial cancer (EC)	hsa-miR-200c-3p [50]	up-regulation (exosome in urine)
Cervical squamous cell carcinoma	miR-221-3p [51]	up-regulation
Bladder cancer	lncRNA (MALAT1, PCAT-1 and SPRY4-IT1) [52]	up-regulation (exosome in urine)
	lncRNA PTENP1 [53]	down-regulation
Urothelial carcinoma of the bladder	Circ RNA circPRMT5 [54]	up-regulation
Gastric cancer	circ-KIAA1244 [55]	down-regulation
	LncRNA HOTTIP [56]	up-regulation
Colorectal carcinoma	LncRNA UCA1 [57]	down-regulation
	miR-6803-5p [58]	up-regulation
Pheochromocytomas (PCCs) and paragangliomas (PGLs)	RET, VHL, HIF2A, and SDHB [42]	mutations
Hepatocellular Carcinoma	mir-21 and mir-144 [59]	up-regulation
	LINC00161 [60]	up-regulation
	mRNA hnRNPH1 [61]	up-regulation
(HCV-related)	lncRNA-HEIH [62]	up-regulation
Female patients	lncRNA Jpx [63]	up-regulation
Liver cancer	tRNA-ValTAC-3, tRNA-GlyTCC-5, tRNA-ValAAC-5 and tRNA-GluCTC-5 [41]	up-regulation

### Exosome lipids in tumor diagnosis

The lipids in exosomes are not only a part of their structure, but their diagnostic value in tumors has been continuously investigated in recent years. A recent study found that there are significant differences of phosphatidylserine (PS) 18:1/18:1 and lactosylceramide (d18:1/16:0) in exosomes between prostate cancer patients and healthy individuals. Furthermore, combinations of these lipid species and PS 18:0–18:2 distinguished the two groups with sensitivity of 93% and specificity of 100% [64]. One study found that the levels of 27-OHC in exosomes from ER+ breast cancer cell line (MCF-7) were significantly higher than exosomes derived from estrogen receptor (ER-) breast cancer cell line (MDA-MB-231), other control exosomes (non-cancerous cell line HEK293 and human pooled serum) by employing capillary liquid chromatography-mass spectrometry. However, the oxysterol profile in exosome did not reflect the cytoplasmic oxysterol profiles of the origin cells, in which cytoplasmic 27-OHC was low in ER+ MCF-7 cells and high in MDA-MB-231 cells [65].

## Exosome enrichment methods

Exosomes do not exist alone in nature, as they often coexist with cell debris, proteins, lipids, and nucleic acids in the blood and cell supernatant. Non-destructive isolation of exosome from complex biological fluid while preserving their structure and function integrity is an indispensable step for downstream exosome analysis. Webber et al. proposed that 3 × 10^10^ EVs per μg of protein indicated high purity of EVs [66]. The main challenge in isolating exosomes comes from their small size. The current mainstream isolation methods are classified into five groups [67] which include differential ultracentrifugation-based techniques, size-based techniques, immunoaffinity capture-based techniques, precipitation, and microfluidics-based techniques. Many literatures [67, 68] have detailed the various isolation techniques, and performance parameters such as exosomes recovery efficiency, assay time and sample volume, bulky instrument. In this section, we have surveyed the recent progress in exosome isolation technology.

### Size-based exosome isolation methods

#### Gel exclusion chromatography

Gel exclusion chromatography is a technique that separates the sample by particle size. It often uses Sepharose 2B or CL-4B to pack the column, then every fraction was collected for subsequent purification. Size based gel exclusion chromatography is found to work well in isolating exosome from contaminating plasma proteins and high-density lipoproteins (HDL). A recent study employed the size exclusion chromatography to extract exosome from the blood, and it showed that the exosomes have good purity [69] with low yield. Moreover, studies also showed that that the exosomes isolated from gel exclusion chromatography have higher biological function compared to that of ultracentrifugation [70].

#### Ultrafiltration

Ultrafiltration (UF) uses ultrafiltration membrane with different aperture to isolate exosomes from protein and other biological macromolecules, and exosomes can be enriched on the ultrafiltration membrane after centrifugation [71]. The commonly used pore size ranges from 1 to 100 nm [72], and the solid the adhesion is, the harder the elution. Hence, drawbacks of UF include challenges in washing away contaminating proteins and elution of exosomes from the filtration membrane. All the above directly negates the yield and purity of exosome. The coated (hydrophilized) membranes can enhance the filtration efficacy to some degree. Merchant et al. [73] utilized microfiltration to isolate human urinary exosomes and found that microfiltration was comparable to UC and will preserve the integrity of exosome structure.

#### Deterministic lateral displacement (DLD) pillar arrays

Wunsch et al. [74] developed nanoscale DLD (nano-DLD) arrays which can accurately isolate exosome from 20 to 110 nm based on silicon chip. When the particle injection stream goes through the array, particles with different sizes will travel in different trajectories, in this case, for a given gap size between pillars, particles with different diameters display different migration angles. Particles with diameter D_P_ (particle diameter) ≥ D_C_ (critical diameter) will be displaced laterally across an array in a bumping mode, with a maximum angle. Particles with D_P_ < D_C_ follow the laminar-flow direction in a zigzag mode, with a mean angle of zero with respect to the array. This method demonstrated its high throughput and high resolution in small size particles isolation. However, it is inevitable that the virus and lipoprotein with the same size as exosome will be co-isolated in complex blood.

#### Viscoelasticity-based microfluidic system

**Fig. 1:**
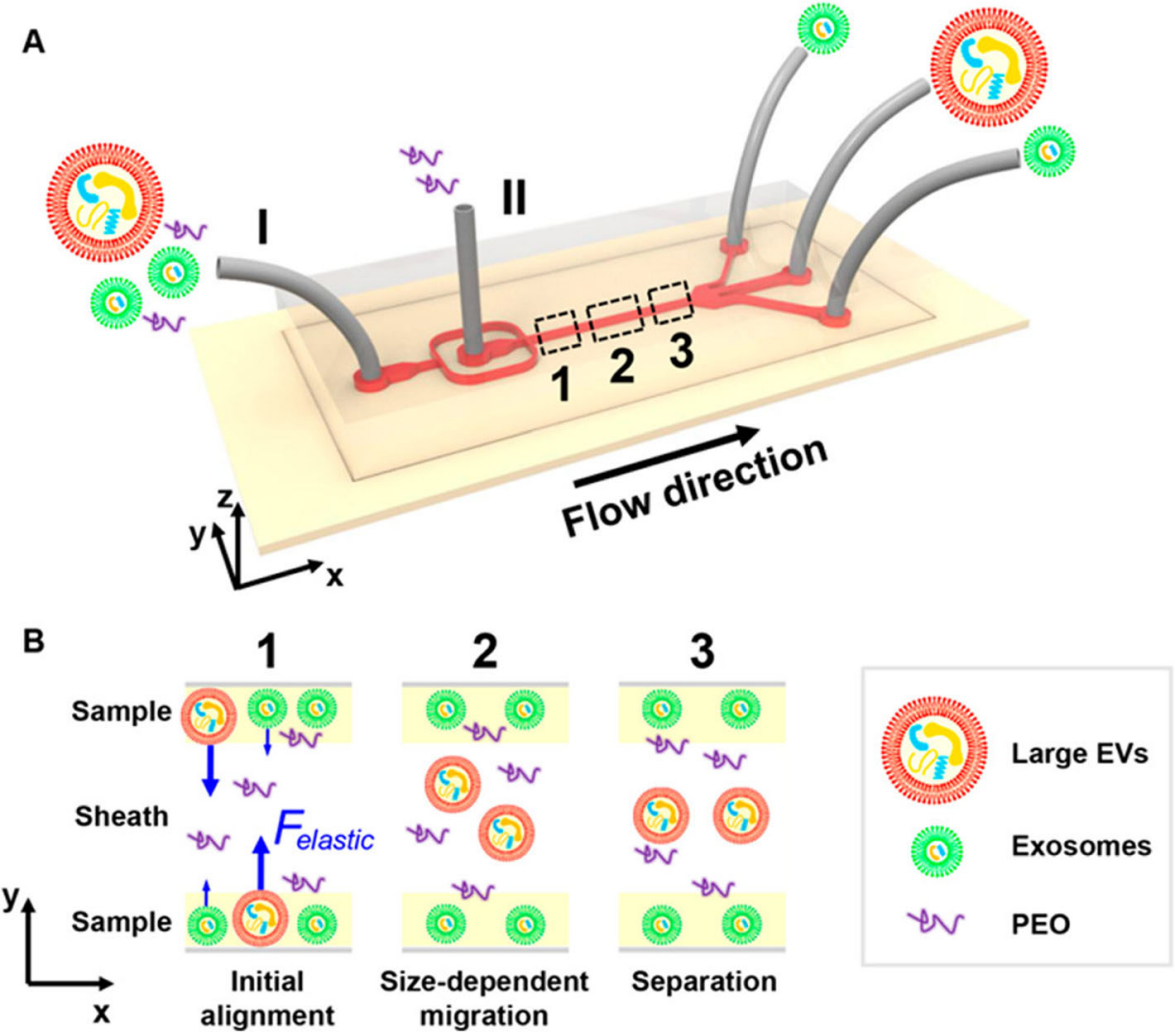
The microfluidic chip for exosome separation from large EVs [75]. Copyright© 2017, American Chemical Society

This is a challenge to separate exosomes from other vesicles such as microvesicles. Liu et al. [75] showed one method which is mainly based on fluid viscoelasticity from PEO (polyethylene oxide). This method can move exosome and large EV to microchannel centerline at a size-dependent rate. The separation mechanism is shown in the Fig. 1. It combines the advantage of both microfluidics and hydromechanics, and this isolation method achieved a high purity (> 90%) and recovery (> 80%).

#### Acoustofluidics-based isolation method

**Fig. 2:**
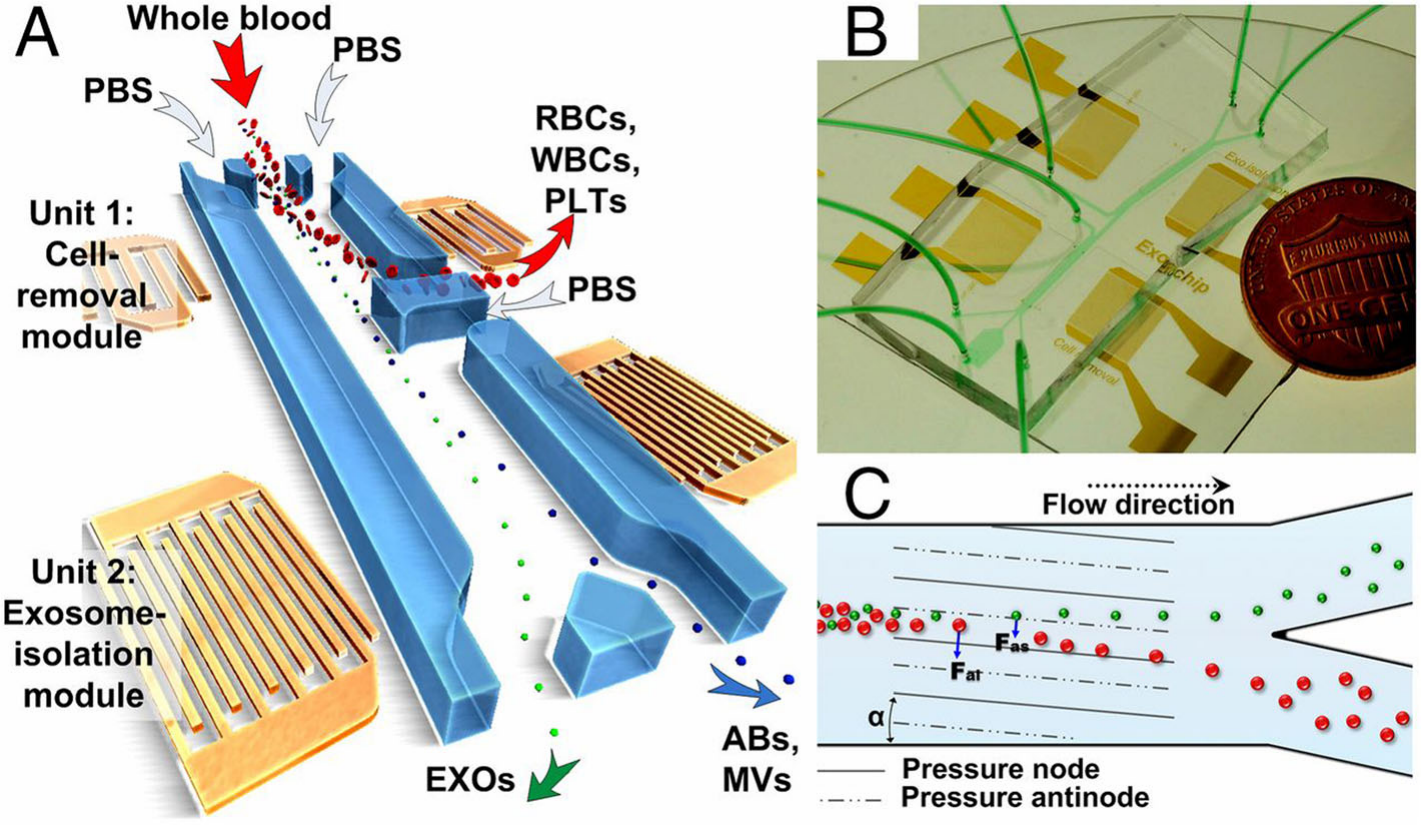
The platform underlying integrated acoustofluidic device for isolating exosomes [76]

The platform [76] is a combination of acoustics and microfluidics that directly isolate exosomes from various biological fluids. As shown in Fig. 2, this device is consisted of two surfaces acoustic wave (SAW) microfluidic modules, respectively achieving the function of cell removal and exosome purification. Its isolation mechanism is that radiation force (Fr) generated by the SAW field and Stokes drag force (Fd) are proportional to the size of particles or cells. For larger particles, Fr dominates over Fd, making them migrate towards the tilted nodes. By continuously adjusting the input power, the suitable cutoff size for exosome isolation can be obtained. When isolating exosomes from extracellular vesicle mixture, the platform can obtain a purity of 98.4%, while isolating exosomes from whole blood can remove 99.999% blood cell. The advantages of this platform are rapid, biocompatible, label-free and need no contact.

### Affinity-based exosome isolation methods

Affinity-based isolation methods often use specific agent that bind strongly to exosome surface marker. The affinity method achieves the merit of higher purity over other physical properties-based methods. Differing from conventional beads, the column [77] and paper [78] are able to be served as capture carriers. Tetraspanin proteins like CD63 and CD9 are often chosen as selection tag for such methods. Apart from the well-established antibodies, other biologically active substance like aptamers [79, 80], lipid probe, heparin [81], and lectin [82, 83] have also been employed in design of exosome affinity-based isolation method. The main technologies are summarized in this section.

#### Immune affinity capture (IAC)

The immune affinity capture technique employs specific antibodies that bind to the surface protein on exosomes. Currently, antibodies have been combined with some new functional nanomaterials and a series of new immunoaffinity isolation techniques have been developed. Apart from magnetic and latex beads, the most commonly used immobilization tools for antibody coating [84] include [78] highly porous monolithic silica microtips [85], graphene foam [86], superparamagnetic nanoparticles [87] and temperature-responsive magnetic nanoparticle [88] to isolate exosomes. It is reported that IAC is the most effective method, and this study shows that the specific marker in exosome isolated by IAC is more than 2-fold higher than that of UC and gradient centrifugation [89]. However, IAC method has high possibility to miss the exosome subpopulations with low expressed surface proteins. To maximize the capture efficiency of IAC [90], we might use a cocktail of the antibodies (such as CD9, CD81, and CD63) to target the surface proteins on exosomes.

#### Aptamer-based isolation method

The aptamer-based method has two forms, an oligonucleotide sequence or a short polypeptide. Aptamer recognizes and binds to their targets like antibody with high specificity and affinity, and have been employed in constructing affinity-based isolation of exosomes. For example, a coating agent consisted of EpCAM-affinity peptide aptamer (Ep114) and zwitterionic poly-2-methacry loyloxyethyl phosphorylcholine (MPC) polymer has been developed for exosome isolation [79]. This material was coated on silica or polystyrene surfaces, which allows capture of EpCAM (+) exosome. The group of Wang et al. [91] utilized MB@SiO_2_@Au nanoparticles decorated with CD63 nucleic acid aptamer to capture exosomes in plasma from cancer patients. Similar studies include use of Vn96, [92, 93] a peptide aptamer has affinity to heat shock proteins (HSP) to capture EVs that express HSP [80]. The study shows that the Vn96 based method obtained higher yield than of UC [93]. Many other peptide aptamers, such as A8 and A17 bind to the different domain of HSP70, peptide aptamer MARCKS-ED and bradykinin (BK) trimer bind to PS [94], peptide aptamer LXY30 targeted α3β1 integrin has been used to develop exosome isolation technology. All these exosome isolation method might have high potential to isolate specific tumor-derived exosome [95, 96]. Due to its high binding affinity toward the protein marker on the surface of tumor-derived exosomes its thermal stability, and commercial availability, the aptamer-based capture methods might have higher potential in exosome isolation compared with antibody-based capture method [97].

#### Lipid-based nanoprobes (LNP) isolation method

Rapid magnetic isolation of EV via lipid-based nanoprobes (LNP) is a method that uses NeutrAvidin (NA)-coated magnetic sub-micrometre particles to capture lipid probes [DSPE-PEG, biotin-tagged 1,2-distearoyl-sn-glycero-3-phosphethanolamine-poly (ethylene glycol)] labeled exosomes, which could isolate exosomes in only 15 min from both of the tumor cell culture or fresh plasma [98]. The highest isolation efficiency is 48.3% for the whole blood sample. Different from immunoaffinity, this separation method relies on pre-modified lipid probe rather than the exosome-specific membrane protein for exosome enrichment. This method can obtain the exosomes with equivalent purity and quality as ultracentrifugation, but without the need for hours of time and bulk of equipment. The yield of exosome has been determined to be feasible for subsequent DNA and RNA analysis.

#### Ligand-based isolation method

Similar as antibody-based affinity capture methods, ligands against specific proteins on the surface of exosomes can also be used to construct affinity-based capture tool for exosome isolation. For example, TIM4 (T-cell immunoglobulin- and mucin-domain-containing molecule) [99] is a protein that bind to phosphatidylserine (PS) in calcium-dependent manner. PS is rich on the surface of exosome [100]. Takeshi et al. modified the magnetic beads with TIM4-Fc as capture reagents. As a result, the method achieved rapid exosome isolation with 4 h. The captured exosomes can be eluted via a chelator such as EDTA, which might hamper the downstream analysis of DNA and RNA. Enzyme-linked immunosorbent assay (ELISA) analysis suggested this method has higher recovery than that of CD9, CD81, and CD63 antibody coated microtiter plate [100, 101]. Another commonly used capture reagent against PS is annexin V [102]. Its binding to exosomes depends on the presence of calcium ions, and exosome will be eluted in EDTA solution. Heparin is a kind of mucopolysaccharide that block interaction between tumor cell EVs and recipient cell [103]. Heparin-conjugated agarose beads can be used for exosome purification from cell culture media and human plasma using ultrafiltration (UF). The method can reach a recovery of 60%. Leonora et al. [81] described a serials of exosome proteins that have unique matched peptides, and these peptides are likely to be explored in exosome isolation in the future.

Lectin is a carbohydrate-binding protein that binds glycan on glycoproteins weakly but with high specificity. Recently, STL lectin (*Solanum tuberosum* lectin) was used to isolate exosome from urine [82]. Exosomes isolated according to different tags differ in characteristics. Studies found that vesicles isolated by antibody and lectin exhibited distinct variations in size and surface content [83]. And some studies found that antibody-based isolation methods may destroy the integrity of exosome since the binding affinity is too strong [101].

### Charge properties-based methods

#### Alternating current electrokinetic (ACE) microarray chip

**Fig. 3:**
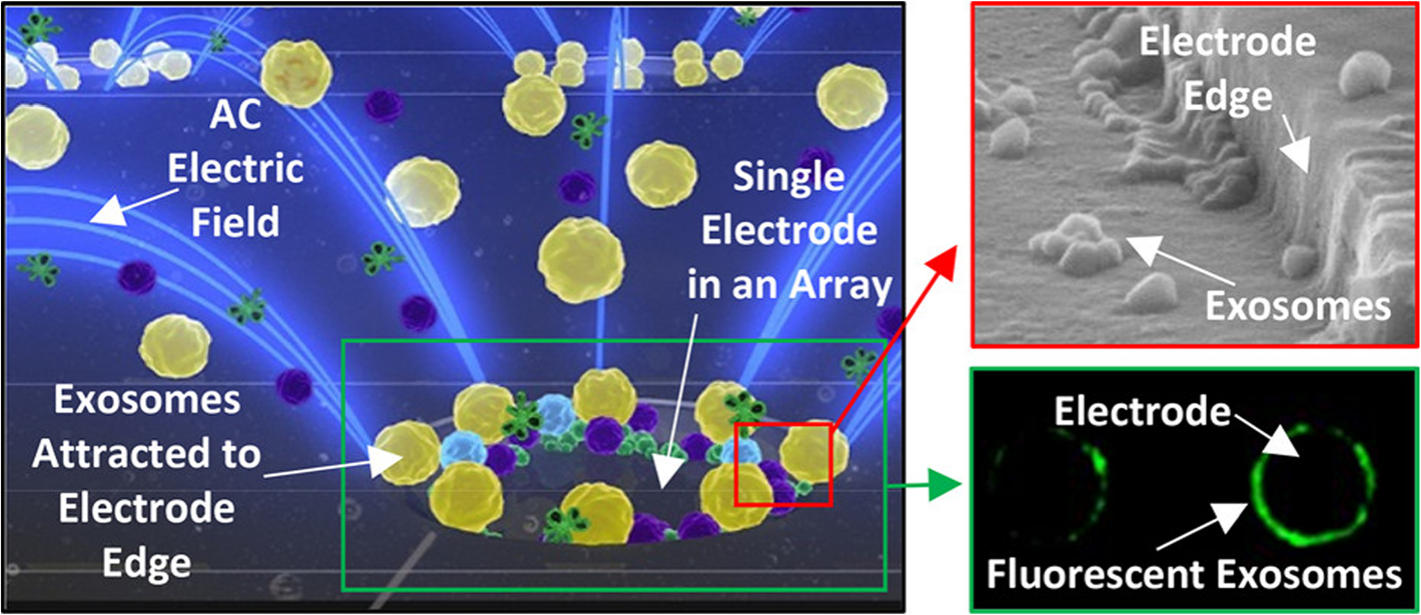
ACE chip microelectrodes collect exosomes and other microvesicles [104]. Copyright© 2017, American Chemical Society

In the isolation force formed by alternating current electric field [104], exosomes and other EVs were pulled in high-field region based on the difference of dielectric properties among different nanoparticles and surrounding fluid. With simple wash, exosomes can be purified from the complex blood sample. Exosomes and other EVs are collected in DEP high-field regions around the edge of microelectrodes. Other large non-EVs components are concentrated in DEP low-field regions between the microelectrodes, which can be washed away and removed. The basic principle is shown in Fig. 3. This technique can directly concentrate and analyze exosome from untreated blood in only 30 min with 30–50 μL sample.

#### Anion-exchange (AE)-based isolation method

Phosphatidylserine (PS) on the surface of exosome membrane is negative charged [105]. Based on this characteristics, Chen et al. [106] used AE magnetic beads to directly enrich exosome in plasma. During the exosome isolation, negatively charged exosomes bind with positively charged AE magnetic beads, while impurities like cell debris, large particles and other positive charged protein will be washed away. It is reported that this method can achieve over 90% recovery efficiency and less protein contaminant than that of ultracentrifugation.

A good exosome isolation method should be compatible with diverse sample matrices and have high exosome recovery with high purity and yield. Multiple encouraging progress has been made in exosome isolation in the presence of overlap in chemical, physical and biological properties between exosome and other extracellular vesicles. All the isolation methods mentioned in the section are summarized in Table 3. The development of ideal isolation technique remains to be a big challenge. Co-isolation of lipoproteins with exosomes is particularly a problem for many sizes or density-based methods in blood plasma samples [116]. Lipid droplets from ruptured cell should be taken into consideration when those surface proteins not specifically expressed on exosomes were chosen for purification. Currently, ISEV indicates that there is no single best isolation method, and they recommend the choice of exosome isolation method will be based on downstream applications [117]. In the future, those platforms which can integrate various exosome isolation techniques for subsequent analysis will substantially increase efficiency for exosome detection.

**Table 3:** Comparison of different exosome isolation methods

Method		Time	Advantages	Disadvantages
Density based methods	Ultracentrifugation [107]	130 min	Relative high purity, allowing exosome isolation in large volume sample	Time consuming, bulk instruments, high speed rotation may cause deformation of exosomes.
	Density gradient centrifugation [108, 109]	250 min	Relative higher purity, can exclude some other EVs.	high requirement for the control of centrifugal time, centrifugal medium preparation is complex.
Precipitation methods	ExoQuick™ and Total Exosome Isolation™ [110–112]	14–16 h	Simple protocol, compatible with a variety of specimens.	time-consuming, low purity, co-precipitation of impurities such as soluble protein
Size based methods	Ultrafiltration [73, 113]	140 min	Simple protocol and time-saving	Exosomes’ blocking or adherence to the filter membrane holes may cause the loss of yield. The force applied to promote the filtration may lead exosome damage, out of shape.
	Gel exclusion chromatography [69, 110]	6–12 h	Simple operation, preserve integrity of exosomes	bulk instrument, relatively low scalable
	Deterministic lateral displacement (DLD) pillar arrays [74]	12 nL/h	High resolution, flexible particle size separation range, no particle labelling, small sample volumes	Complex parameter settings, low operability, pre-purification needed, relative high risk of clogging
	MicrofluidicViscoelastic Flows [75]	200 μL/h	High purity (> 90%) and recovery (> 80%), field-free, label-free, fast, low cost, cutoff size is regulatable.	PEO is hard to remove and may influence subsequent analysis
	Acoustofluidic [114]	∼25 min	Direct separation from biological fluids label-free, high yield and purity, cutoff size is flexible, automation, high reproducibility,	Aggregation of lipids in blood may greatly reduce separation efficiency.
Affinity isolation methods	Immune affinity capture [89]	240 min	high purity, milder manner for exosome isolation, preserve structure integrity of exosome.	overlook the subpopulation without affinity marker, non-specific binding, not suit for large scale exosome purification
	EpiVeta [79]	>10 h	Peptide aptamer is versatile and easier to prepare. This coating layer can be combined with a variety of solid phase carriers.	Specimens require pre-processing and the process takes a long time, lacking verification of body fluid exosome.
	Lipid nanoprobe (LNP) [98]	15 min	Fast, high yield, compatible various downstream analyses of DNA, RNA and proteins.	lack specificity, other lipid and albumin in blood could be co-purification, magnetic bead separation may cause the shrinkage of nEVs
	TIM4-Fc-conjugated beads [101, 115]	4 h	high purity, preserve function of exosome.	purification efficiency decreases when the volume of the sample is over 1 mL and TIM4. inhibitors (EDTA and citric acid) existed, The separation step is complicated and requires pretreatment, yields vary greatly among different sample.
Charge properties based methods	Alternating current electrokinetic microarray chip [104]	<30 min	Direct separation from plasma, label-free, in situ detection, fast	possible contamination of protein polymers with similar charging properties
	anion-exchange (AE)-based isolation method [106]	30 min	direct separation from plasma, high recovery efficiency (> 90%), fast, high purity.	Varying salt ion concentration may affect the structure and function of vesicles while elution, possible contamination of protein polymers with similar charging properties

## Exosome quantification methods

As mentioned above, the absolute amount of exosome in bodily fluid directly suggests the presence and stage of cancer. There is a variety of techniques currently available for exosome quantification. And there is no consensus that which method is the best option. Exosome quantification can be categorized into two different methods: unspecific counting methods and general quantification methods which are based on common substances in interested exosomes. Unspecific counting methods often obtain an absolute value that can be compared between different studies. Those methods often perform direct counting exosomes one by one based on their physical properties, like optical. It is mandatory to do pre-isolation before analysis. In terms of tumor derived exosome quantification, these widespread substances often refer to various markers with diagnostic value for multiple tumors, like protein, ribonucleic acids etc., as mentioned before.

### Unspecific counting methods

Unspecific determination methods only obtain a rough estimation of the number of vesicles present in sample, and they are limited by primitive purification prior to analysis and various detection threshold setting. Currently unspecific counting techniques include Nanoparticle Tracking Analysis (NTA) [118], Resistive Pulse Sensing (RPS) [119], Tunable resistive pulse sensing (TRPS), Dynamic light scattering (DLS) [120, 121] and electron microscopy (EM). The principle, potential advantages and disadvantages of each methods have been discussed and summarized in several reviews [122, 123]. 2017 methodological guidelines [68] from ISEV compared estimated count rate and detectable size range in NTA, RPS, flow cytometry, and EM. Among them, the guideline found out that flow cytometry is able to quantify the number of exosomes and record specific fluorescent signal as particles pass though, and their size can be calculated from the side scattering signal [124]. The mechanism of nanoparticle flow cytometry is almost the same as flow cytometry. In brief, when the particles travel through the fixed laser beam, the nanoparticles would scat the light, and the size distribution would be obtained by analyzing these light signals. Many scientists have focused on in down-regulating detected level of particle size. Owing to relatively small size of exosomes, the light signal difference between the background noise and target particle is quite subtle. Theoretically, lower laser wavelengths can detect smaller particle size. CytoFlex was developed by Beckman Coulter company by introducing violate side scattered light (VSSC) (405 nm) and Fiber Array Photodiode (FAPD) patented technology. It can reduce the detection limitation to 200 nm. Britain Apogee Company’s Apogee A50 Micro [125, 126] can detect about 100 nm nanoparticles, benefiting more from its excellent light optical technology that can discriminate small vesicles from noisy ones. Using polystyrene or silica beads as standard for determining nanoparticle size is not accurate [68], Apogee A50 Micro can also correct results by combining their optical parameters. Ye et al. [12] developed a high-sensitivity flow cytometry with a EV detection range of 40–175 nm, and further reduced the probe volume to 25 fL (femtoliter) and extended the dwell time when nanoparticles pass through the laser beam to ms (milliseconds). As a result, this method effectively decreased the background signal and enhanced emitted photons.

ImageStreamX MKII of EMD Millipore company [124] presented the image of particles in the same manner as the optical microscope, which makes it possible to distinguish exosome and other cell debris. The use of charge coupled device (CCD) cameras in the instrument instead of traditional photomultiplier tubes leads to wider dynamic range and less noise. Although ImageStreamX can detect particles as small as 100 nm with the help of fluorescence imaging, but it is still not possible to direct measure the size of exosomes. Indeed, since fluorescence backgrounds are much lower than scatter, the binding-induced fluorescence can partly resolve this problem [127]. Under the fluorescence to sort activated exosome, not only the sensitivity is improved, but also exosome surface molecules can be simultaneously detected. Double labeling with protein- and lipid-specific dyes enables separation of EVs from common contaminants of EVs preparations, such as protein aggregates or micelles formed by unbound lipophilic styryl dyes, which is able to eliminate overestimation of numbers of EV [85]. Moreover, Groot et al. [128] sorted subsets of EVs differentially labeled with two fluorescent antibodies with high purity by altering nozzle size and sheath pressure. They also found that swarm effects that high concentration particles will severely impair EV quantification and characterization. Multiple objects going through the interrogation point in the same time may be mistakenly counted as one big particles [129]. Therefore, an appropriate concentration with proper flow rate is always needed to ensure a reasonable acquisition rate using flow cytometry for exosome detection.

### Quantification based on exosome content

Proteins present inside of exosomes are inaccessible due to the lipid membrane envelope. Methods in these parts accomplish the quantification by relying on multiple chemical reactions, to transform the tiny vesicles to signals detectable by instrument or human naked eyes. Some of them have integrated the enrichment with quantification, making it possible to perform raw blood analysis. This following section focuses on commercial kits and several remarkable methods developed in the recent years.

#### Quantification by commercial kits

There are a lot of quantification kits based on certain substance in SBI exosome, such as EXOELISA-ULTRA, EXOELISA, EXOCET, FLUOROCET, and EXOCET. These methods are either based on colorimetric (fluorescent) method or ELISA as one of the representative products. This technology is based on the fact that Acetyl-CoA Acetylcholinesterase (AChE) is known to be enriched within exosomes [130, 131] from serum, stem cell, cancer cells, mesenchymal stem cell (MSC) etc. Each exosome is not necessarily to contain an equal amount AChE, so the accuracy of this method might be problematic. Moreover, the blood also contains some AchE, in order to avoid errors, the preparation should be completely washed before detection. Of course, some drug like AchE inhibitors should also be taken into consideration [132]. Moreover, Exo-TEST kit from LONZA company is a double sandwich ELISA assay. The special feature of this method is that foreign antibodies (pan-exosome antibodies) are needed to mediate the adsorption of exosomes and solid phase carriers [133, 134]. Compared with EXOCET, it doesn’t need exosome purification. Based on this principle, the affinity and specificity between foreign antibodies and exosome seem to be quite vital for detection accuracy. Similar kits also include ExoQuant, Overall Exosome Capture and Quantification Assay Kit.

#### Membrane-based quantification approaches

Quantification methods in this section were carried out based on either membrane modification with chemical group or immune recognition of membrane protein by antibodies. To obtain an absolute number of particles per milliliter, the establishment of a standard curve based on NTA is needed.

##### Exosome quantification via bivalent-cholesterol labeled DNA anchor for signal amplification

**Fig. 4:**
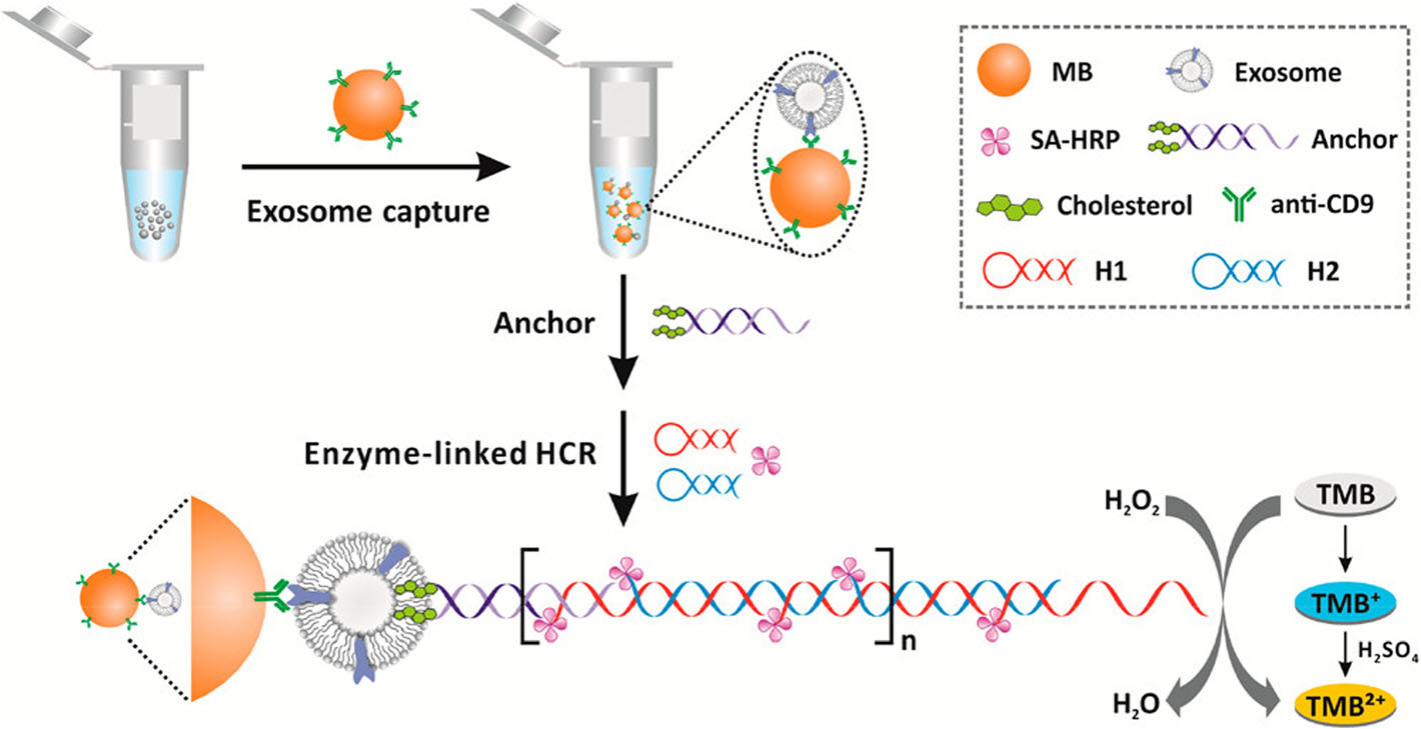
Exosome quantification by a method based on immunoaffinity separation combined with cholesterol signal amplification [135]. Copyright© 2017, American Chemical Society

The principle of this exosome quantification [135] (Fig. 4) is as follows: The exosomes are specifically captured by anti-CD9 immunomagnetic beads and then DNA anchors labeled with high affinity bivalent-cholesterol spontaneously inserted into exosomes. The anchor’s sticky end can trigger a horseradish peroxidase (HRP)-linked hybridization chain reaction (HCR). The detection was based on HRP-catalyzed H_2_O_2_ mediated color changes of 3,3′,5,5′- tetramethyl benzidine (TMB). The method can sensitively detect a concentration of 2200 particles/mL with a relative standard deviation of less than 5.6%.

##### Nanoparticle counting by microscopic digital detection

**Fig. 5:**
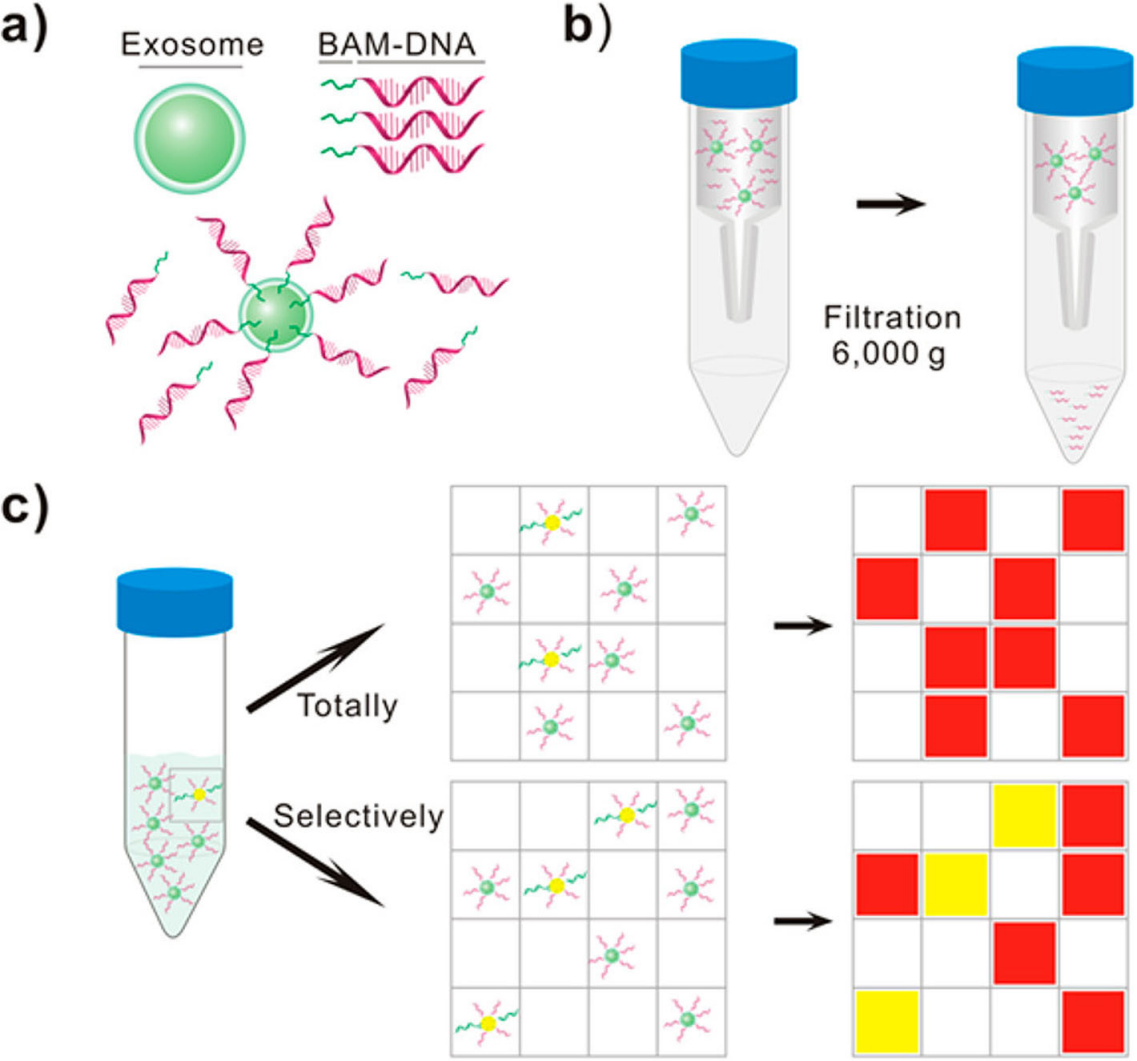
Exosomes counting by microscopic digital detection via surface-anchored nucleic acid amplification [136]. Copyright© 2018, American Chemical Society

This method [136] utilized digital detection to qualify total exosomes and disease-specific exosomes, which is based on nucleic acid amplification in microchip. Mechanism is shown in Fig. 5. The poly (ethylene glycol) oleyl ether (biocompatible anchor molecule, BAM) conjugated with DNA oligonucleotides is anchored to the lipid bilayer membrane of exosomes through surface self-assembly. The specific antibody (glypican 1 antibody)-DNA conjugate binds to specific subgroups in total exosomes. Exosomes are then assigned to each chamber after removal of free DNA by ultrafiltration unit, ensuring each chamber has one or less exosomes. With fluorescence signal amplification, normal cell-derived exosomes and disease-specific exosomes will emit red and yellow fluorescence in the chamber, respectively. By simple digital detection and Poisson distribution, exosome quantification can be achieved. This method can be combined with various types of established nucleic acid analysis, but this method requires advanced purification for exosome.

##### Quantum dot-based exosome quantification

**Fig. 6:**
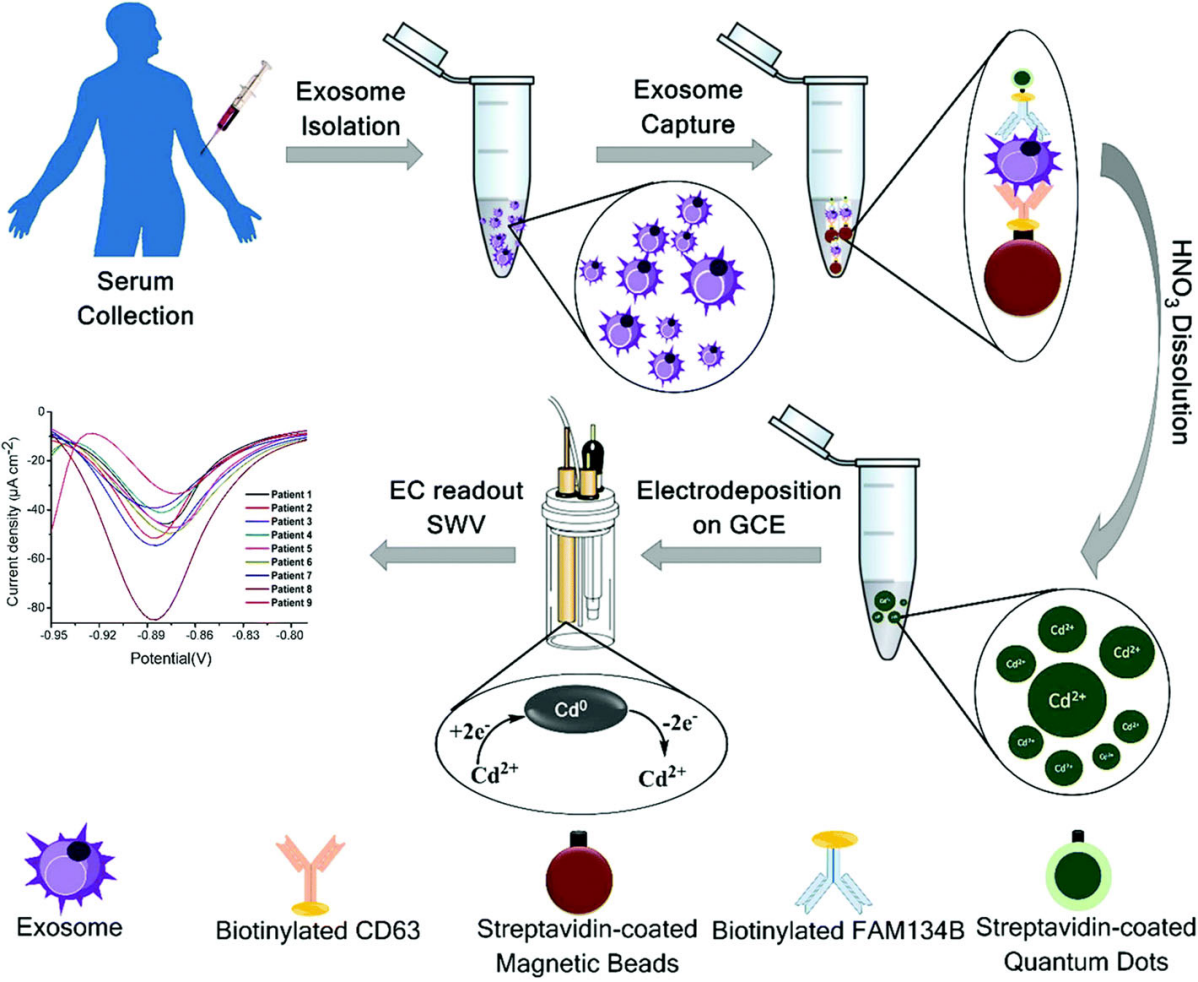
The isolation and quantify method of cancer-specific exosomes based on CdSeQD [137]. Copyright© 2017, Royal Society of Chemistry

Currently, there were some studies using quantum dots to quantify exosomes. As shown in the Fig. 6, Boriachek et al. [137] used exosome-specific antibodies to capture exosomes on magnetic beads, and then used CdSeQD-functionalized specific antibodies to isolate cancer-specific exosomes. Tumor-specific exosomes were quantified by the detection of CdSeQDs. This method used quantum dots as signal amplifiers and combines volt-ampere measurement with immune technology to determine disease-specific exosomes. The detection sensitivity of tumor cell lines derived exosomes can reach 100 exosomes/μL, and %RSD (relative standard deviation) < 0.05. Application of tumor-specific exosome protein antibodies (FAM134B for colon and HER2 for breast cancer) is one of the features of this method, which represented a promising bioassay technique.

##### Droplet Digital ExoELISA

**Fig. 7:**
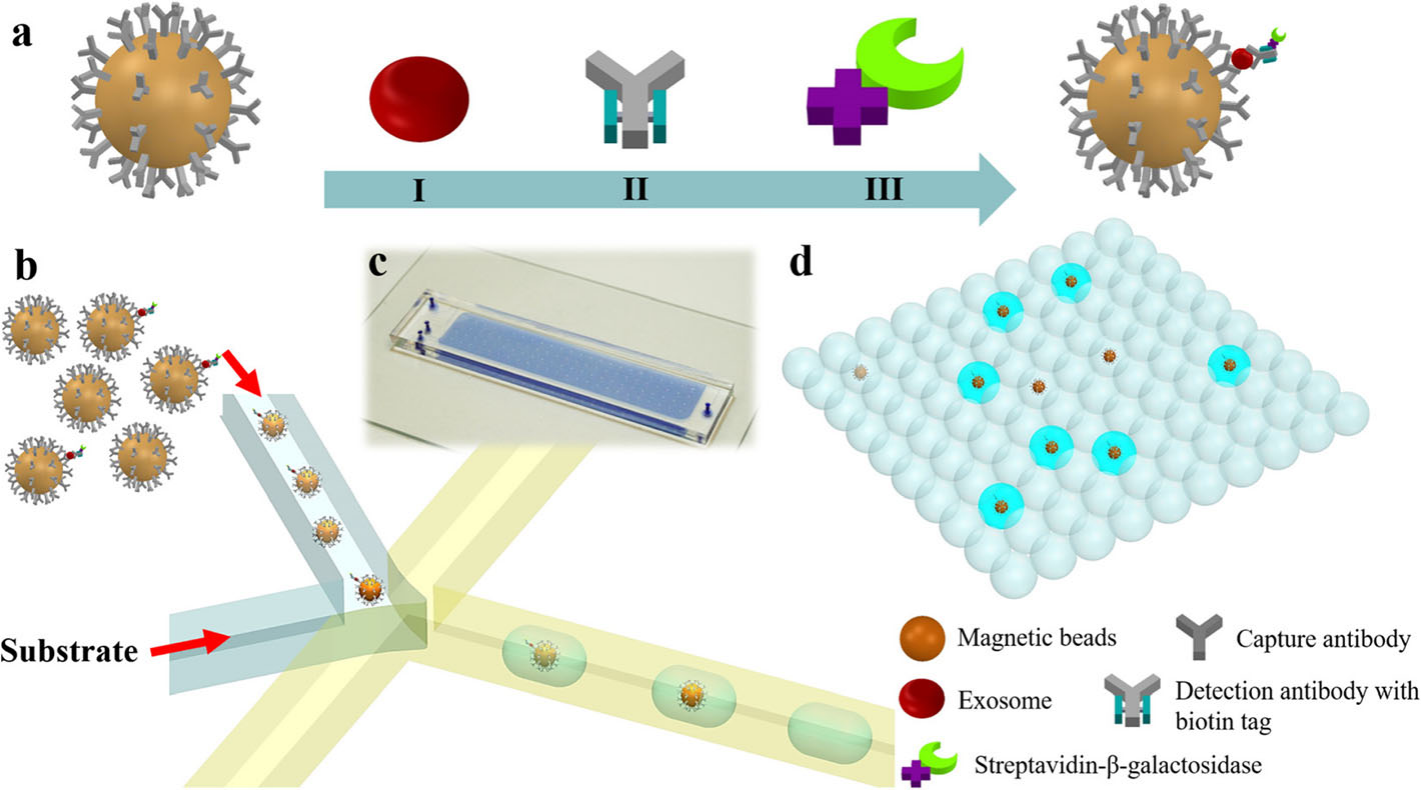
The droplet digital ExoELISA for exosome quantification [138]. Copyright© 2018 American Chemical Society

Recent study showed the droplet digital ExoELISA for exosome quantification [138]. As the Fig. 7 shows, exosomes were captured by CD63 antibody coated magnetic beads. Specific antibody (glypican 1 antibody) conjugated with β-galactosidase which catalyzes the fluorescein-di-β-D-galacto-pyranoside (FDG), and sandwich ELISA complexes, were isolated into sufficient number of droplets to insure only a single bead is present in a droplet. Fluorescence signals represent the presence of exosomes. Their concentration can be obtained after signals statistical analysis. The detection limit of this technique can reach down to 10 enzymes per microliter (LOD) for labeled exosomes (~ 10–17 M), and the linear correlation with nanosight measurement results can reach 0.995. This method selects antibodies to purify exosomes, and there are also leak detection for some CD63-low expression exosomes.

## Exosome contents detection

### Exosome protein detection

Protein is the core component of human metabolism, acting as a break point for the discovery of novel biomarker for tumor diseases. Traditional protein detection methods like western blot (WB) and enzyme-linked immunosorbent assay (ELISA) are not suitable for routine clinical use with bulky specimens, because of their large sample consumption, cumbersome operation, and special instrument. At present, the detection of exosomes is mainly based on antibody, aptamer and proteomics related mass spectrometry. Antibodies have been used to detect proteins for a long time, and with the rise of aptamers, the shortcomings of its preparation become apparent. The detection method using mass spectrometry is too blind and complicated, which makes it is not suitable for rapid and targeted clinical detection in the future. The aptamer detection method for proteins can be combined with mature nucleic acid technology, making it a promising alternative strategy.

#### Antibody-based methods

This following part focuses on a series of recently developed antibody-based techniques for exosome protein profile, and the working principle and their performance parameters for each method will be elaborated. Methods in this part often employ the mechanism whereby reporter molecular conjugated antibody is incubated with exosome antigen, in which the antigen amount is proportional to the intensity of reporter signal. Highly specificity and high affinity of antibody are both two key factors in developing a robust immunoassays [139]. The combination of several antibodies can achieve multiple detection of different antigens in one time, which enhances the efficiency of analysis and diagnostic performance, but the possibility also give rise to false positivity due to unspecific binding in multiplexing assay [140]. At the same time, owing to rapid development of exosome biomarkers, there are no accessible antibodies in the market for these biomarkers. The specific markers of exosome subpopulation that track the parent cell is still a big challenge and need further development. The discovery of such makers will provide more detailed information on tumor location. Some classic immunoassay methods are summarized in Table 4. Therefore, we will pass over the introduction for these methods. Table 5 describes some novel antibody-based detection platforms, which includes their principle, dynamic range, and potential advantages and disadvantages.

**Table 4:** Classic immune analysis techniques for exosome proteins

Method	Basic principle	Signal output	Sample volume (μL)	LOD (particles/mL)	Analysis time ≤ 2 h	Advantages	Disadvantages
Surface Plasmon Resonance (SPR) [141–145]	Binding between EV and sensor surface coated with specific antibody induces refractive index change.	Refractive index	20	10^7^	yes	Label-free, monitor binding between exosome and antibody	require special instrument
Fluorescent Immuno Sorbent Assay (FLISA) [90, 146]	ELISA based method	Fluorescence	1	10^10^	no	High sensitivity	problem of auto fluorescence and fluorescence quenching
Time-Resolved Fluorescent Immuno Assay (TRFIA) [147]	Based on long half-life of europium	Phosphorescent molecules (like europium)	100	10^10^	no	More sensitive than ELISA	europium is harmful for health
Integrated Microfluidic Exosome Analysis Platform (IMEAP) [84, 148]	Combination of MAIA technique and microfluid	Fluorescence	30	10^8^	yes	More capture surface than ELISA, micro fluid improves efficiency	_
Amplified Luminescent Proximity Homogeneous Assay (ALPHA) [149]	EV pulls two beads as close as 200 nm, accepter beads uptake O2 from donor bead after being activated	Emitted light	5	10^10^	yes	High sensitivity and simple reaction system, signal amplification	signal fluctuation and hook effect
Micro-Nuclear Magnetic Resonance (μNMR) [150, 151]	Immunomagnetic nanoparticles binding to EV surface antigen induces magnetic field change	Magnetic susceptibility	1	10^7^	yes	Simple operation	require special instrument

**Table 5:** Comparison of antibody-based analysis technology for analyzing exosome proteins

Method	Basic principle	Signal output	Sample volume (μL)	LOD (particles/mL)	Dynamic range	Analysis time	Advantages	Disadvantages
iKEA (integrated kidney exosome analysis) [152]	Combination of MAIA (Magnetic antibody immunization assay) and chip technique	Electrical currents	0–15,000	1.6 × 10^4^	10^4^	2 h	detection signal in this platform can be wirelessly transferred to Bluetooth-ready devices	The exosome needs to be purified in advance
ExoPCD-chip [153]	CD63 (an enriched marker in exosomes surface) aptamer26 and hemin/LGCD (formed by mimicking DNAzyme sequence and CD63 aptamer) trigger redox reaction of NADP; a Microfluidic technique based on immune magnetic bead.	absorbance	30	4.39 × 10^3^	10^5^	3.5 h	without purification in advance	The reaction system is complex and the detection process takes a long time
ZnO nanowires coated three-dimensional (3D) scaffold chip [154]	utilize ZnO nanowires immobilized with exosome-specific antibody to isolate exosome, and colorimetric assay (HRP catalyze H_2_O_2_-mediated oxidation of TMB) for exosome detection.	absorbance	100	2.2 × 10^4^	10^3^	–	The qualitative result can be observed by naked eyes. Chip is small and without special instrument for result reading. Separated exosomes can be released again	Serum and plasma serum or plasma need to be pumped rather than directly added to.
PDA encapsulated antibody-reporter-Ag (shell)-Au (core) multilayer (PEARL) SERS tags chip [155]	polydopamine-modified immunocapture substrates and an ultrathin polydopamine-encapsulated antibody-reporter-Ag (shell)-Au (core) multilayer (PEARL) Surface-Enhanced Raman Scattering (SERS) nano-tag with quantitative signal of the Raman reporter at 1072 cm^−1^: a sandwich immunoassay	Raman intensity at 1072 cm^− 1^	2	5.418 × 10^2^	10^3^	3 h	ultra-smallsample volume, high sensitivity.	Experimental materials are complex and expensive to construct
ExoCounter [156]	The sandwich structure (Ab-exosome-Ab-conjugated single FG bead) on a removal plate Containing 16 wells on DVD is detected by a photodetector to achieve specific exosome quantification at the removal of optical disc drive.	relative voltage	0.39	about 10^6^	10^3^	2.5 h	Label-free, without pretreatment, higher sensitivity than flow cytometry	Limited by antibody binding force, some exosomes may be missed
Electrochemical assays [157]	Combination of a sandwich immune assay and electrochemistry detection	current signal	5	4.7 × 10^8^	not offer	2 h	Cost-effective, require tedious electrode surface functionalization.	Reproducibility is not good and sensitivity is low

##### Western blot (WB) and ELISA

Western blot, also known as immunoblotting, is based on basic principle that colors the gel-electrophoresis-treated cells or biological tissue samples by specific antibodies. As a golden standard, WB is the most used in EV research to validate the presence of exosome in purified preparation via its characteristic surface proteins (CD9 and CD63). Processing by lysis solution contains protease inhibitor, exosome solution is then separated by sodium dodecyl sulfate polyacrylamide gel electrophoresis (SDS-PAGE) [158], which is then incubated with primary antibody and secondary antibody after transferring to the membrane. WB provides the information on molecular weight of target protein.

ELISA is another commonly used method for qualitative and quantitative protein detection based on antigen-antibody specific binding. As a classic method in immunology, it can be performed in multiple formats, like sandwich method, indirect method, and competition method. Compared with WB, ELISA is faster, easy to handle, more likely to adapt to throughput manner, but it has large variability.

##### Alternating current Electrokinetic chips

This technique [159] pulls nanoparticle like exosomes to the edge of a tiny electrode from other complex blood substance while on alternating current. Large cell and debris will then be washed away with exosome left behind at the effect of alternating electric field. This step can be completed in only 20 mins, with only 25 μL plasm or serum without any dilution. Scientists add specific antibody targeted to CD63 or glypican-1 (markers of pancreas ductal carcinoma) labeled with fluorescence. Bright color circle is formed by antibody binding to exosome distributed around microelectrode after incubation and washing, which can then be seen under the microscope once the CD63(+) or glypican-1(+) exosomes exist. The total time takes less than 1 h. In this study, the detection limitation of the chip can go down to 3.3 × 10^9^ particles/mL. The advantage of this method is short and easy protocol, and can also be applied to primary screening in clinical setting. However, this method still cannot eliminate the contamination of lipid protein.

##### intravesicular nano-plasmonic system (iNPS)

Currently, most detection methods are limited to exosome surface protein, but this EV screening assay [160] can in advance detect both intravesicular (AKT1) and transmembrane protein (EpCAM, CD63) of exosome via lysis. This system relies on nanohole-based surface plasmon resonance (SPR) technique. The chip is formed nanoholes with a diameter of 200 nm in a thin (100 nm) golden film. The chip surface is coated with specific antibody as ELISA, and an obvious signal shift will be detected once the double antibody sandwich (antibody-protein-antibody-AuNPs) forms. In this platform, only 0.5 μL of sample is required for each marker, almost 200-fold volume of sample less than of ELISA.

##### Raman tweezers microspectroscopy (RTM)

RTM has been used to characterize exosome chemical composition (relative amount of nucleic acids, lipids and proteins) via Raman fingerprints, which could be completed in several seconds or minutes without any label. Zachary et al. [161] used the optical tweezer method and found that spectral variation may origin from cholesterol and protein expression in exosome surface. Moreover, Ire’ne et al. [162] attempted to detect human urine exosomes by RTM. It should be noted that the exosomes in this study needed to be purified from urine. Randy et al. [163] combined multispectral optical tweezers (MS-OTs) and fluorescence antibody labeling to make Raman spectra measurement of CD9(+) exosome subpopulations. The labeled and fluorescent exosomes were trapped with 785 nm optical tweezers. Compared with other more informative methods such as proteomics, genomics, optical tweezers combined with Raman spectroscopy technique may not provide comprehensive data on protein and nucleic acids in exosomes, but it can serve as complementary technique for those other time-consuming method. In summary, it is a promising alternative method for rapid exosome characterization.

#### Aptamer-based methods

It has been widely known that the antibody can be employed as capture tool for exosomes isolation. However, recent reports suggested that the single-stranded oligonucleotides possess similar binding affinity with specificity for associated molecules on the exosome membrane.

##### Multiple detection of exosomes using magnetic substrates and SERS probes

**Fig. 8:**
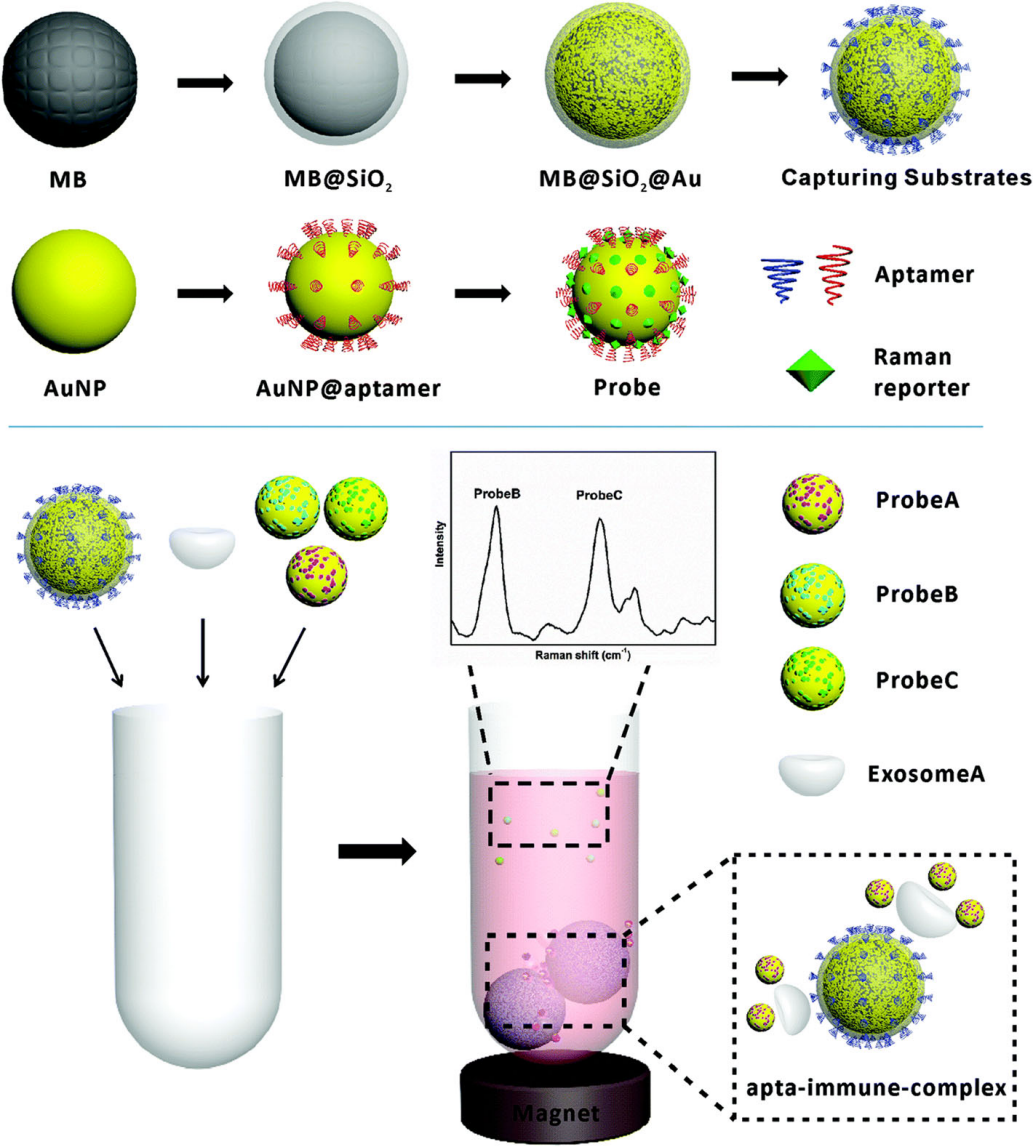
The principle of SERS-based detection method for exosomes [171]. Copyright© Royal Society of Chemistry

Surface enhanced Raman spectroscopy (SERS) is a technique derived from Raman spectroscopy. Raman spectroscopy is an optical technique that is based on detection of inelastic scattered light when a particle is illuminated by monochromatic laser light. The energy transportation related to molecular vibration will induce a wavelength shift, which can served as a specific footprint for different molecules [164, 165]. Raman spectrum can be used for exosome size measurement or quantification as well [166]. Since trapping process in Raman spectroscopy analysis is a random process, overlong measurement time strongly hinders its application [167]. Meanwhile, the too subtle signal from exosome become another obstruction. So here comes the SERS technique. Raman signal can be strongly enhanced in SERS (up to 10^14–15^ times). It is based on plasmon excitation on irregular metal surfaces, usually, Au or Ag. SERS can serve as a valuable tool to discriminate exosome subpopulations [168, 169]. SERS technology has been widely used in ultrasensitive detection of exosomes, whether quantification or characterization [155, 170]. This method uses magnetic substrate and SERS (surface enhanced Raman scattering) probe to detect multiply exosomes. As principle is shown in Fig. 8, firstly, universal surface protein CD63 aptamer-modified gold shell magnetic nanoparticles are used for exosomes capture. Three gold nanoparticles, as probes, are respectively modified with aptamers (CEA for colon cancer, H2 for breast cancer, PSMA for prostate cancer) targeted specific exosomes and three Raman reporters (DTNB, MMC, and 2NAT) are then simultaneously added into above magnetic complex. With the formation of golden particle-positive exosome-magnetic beads complex, the decreased Raman signal peak is detected in the supernatant after magnet separation, showing the presence of cancer-specific exosome. For exosomes from SKBR3 cell (breast cancer cell), the LOD values can reach down to 32 exosomes per microliter and dynamic range can reach four magnitude [171].

##### Aptamer/AuNP biosensor for colorimetric profiling of exosomal proteins

**Fig. 9:**
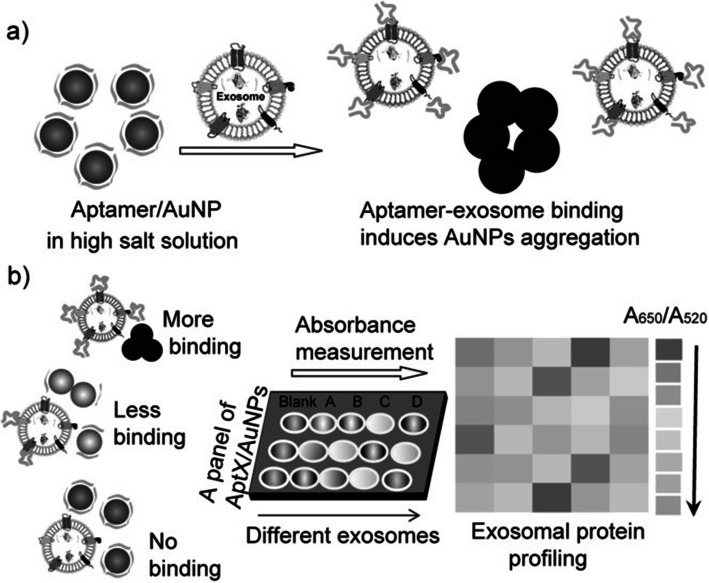
The aptamer/AuNP complex used for molecular profiling of exosomes [172].© 2017 Wiley-VCH Verlag GmbH & Co. KGaA, Weinheim

This method [172] involves visual detection of exosome surface protein. This platform utilized aptamer on AuNP and protected its aggregation in high-salt solution. But when special exosome appears in the sample, stronger binding between aptamer and exosome separates the aptamer from AuNP, forming visual deposit. The principle is shown in Fig. 9. The method achieves profiling via a panel of aptamer/protein interactions successively, not protein scanning in the true sense.

.

##### SOMAmers platform

SOMAmers (Slow Off-rate Modified Aptamers), sometimes referenced as SOMAscan Array, is formed with high affinity (10^− 9^ to 10^− 12^ M) and high specificity chemically modified aptamer to target protein. With multiple aptamers assembling in a small platform, this device can precisely measure more than 1100 proteins, but has the same performance as sandwich ELISA in sensitivity (LOD 40 fM). This technique has been engaged in discovery of cancer associated marker protein [173]. Jason et al. [174] utilized SOMAscan™ array (version 3.0) to detect Du145 prostate cancer cell line derived exosome protein profiling. They found more than 300 unknown exosome protein previously, suggesting SOMAmers based technique is an effective weapon for exosome protein profiling. Moreover, this technique is also used for serum, plasm, tissue lysis and cerebrospinal fluid [175, 176]. However, for most other antibody-based platforms, arrays are limited to less than 100, with the interference of second antibody to reaction specificity, making them not very efficient compared to SOMAmers platform [177].

#### Proteomics analysis with mass-spectrometry (MS)

Proteomics analysis of exosomes was firstly applied to dendritic cells derived exosomes in 2001 [178]. Early MS can only detect high-abundance exosome protein. The MS technique can provide complete information about protein profile of exosome, which is more likely to find new biomarkers for disease diagnosis and other functional proteins. To date, more than 1000 exosome proteins in urine were identified via MS [179] Generally speaking, there are two paths that can be used to analyze exo-protein: one involves removal of surface protein with maintenance of intact structure of exosomes, and the other uses lysis agent to disrupt the whole spatial configuration of exosome, causing total protein distribution in the solution. The shaving of exosome surface protein need to remove post-translational modifications, purify protein by filter-aided sample preparation (FASP) method with artificially added enzyme and other agents [110] like trypsin [180]. A review [181] paper has summarized the present methodological approaches for high-throughput mass spectrometry-based proteomic analyses of exosomes. SBI company has developed the XPEP kit to cleave away of protein from exosome surface. Of course, the peptide library obtained from exosome total lysis stand more for protein composition and contribute to biomarkers discovery of inner protein, considering the fact that surface protein only take in 20% of the total protein content [110]. Current standard instruments for exo-protein analysis conclude nano LC/MS/MS Q Exactive of Thermo Fisher with Waters Nano Acquity HPLC system, while sequent peptide identities need to be mapped to Mascot databases. There are several points that need to be remembered in mind: The MS for protein analysis has strong randomness since there is a step for enzyme digestion. Sometimes, owing to its high sensitivity, the specificity from MS is correspondingly decreased. Despite use of cell line medium, clinical serum, or dedicated bioreactors, the soluble protein released by cells in MS is very hard to eliminate, making high requirements for exosome purity preparation [182–184], making the already complex steps more cumbersome. And considering its low repeatability, the method is not suitable for clinical application. As for data analysis, the group and classification of detected proteins should be compared with an authoritative database like Vesiclepedia [185], Exocarta, EV pedia [186].

### Exosome nucleic acid detection

**Fig. 10:**
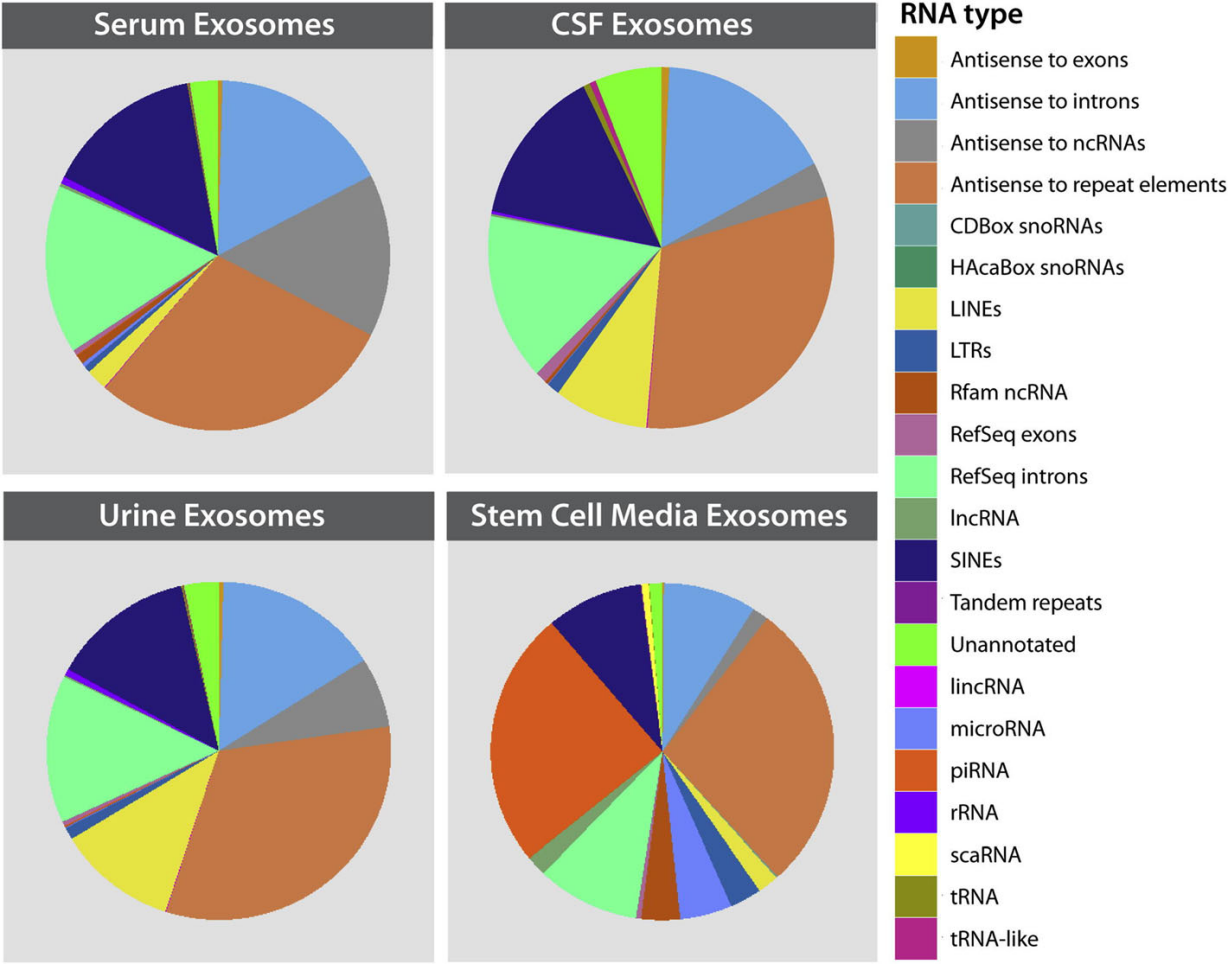
The abundances and types of specific RNA classes present in exosome by NGS sequence [110]. Copyright© 2015 Elsevier Inc. All rights reserved

Emerging reports have asserted exosome indispensable function in intercellular communication, as exosome RNA has key role among all exosome cardo. Figure 10 shows RNA types in exosome of various origins [110]. The potential of exosomal RNA in clinical diagnosis and therapy warrants application of more advanced techniques for exosomal RNA analysis and RNA composition comparison between the cancer-derived exosome and normal exosome.

After purifying exosomes from plasma or cell culture supernatants via suitable isolated method, RNA can then be extracted by purification kits, such as SBI’s SeraMir kit, mirRCURY RNA Isolation Kit (Exiqon, Vedbaek, Denmark) [187], Exosome Total RNA Extraction Kit (HansaBioMed), phenolisopropanol precipitation (Trizol, Invitrogen) or Exosome RNA Isolation Kit (Norgen Biotek). However, the isolation methods for exosome will actually affect RNA measurements to a certain extent [188]. If the blood sample comes from the heparin anticoagulant tube, it is recommended to treat the plasma with heparin enzymes to prevent potential interference in subsequent reverse transcription experiment [110].

RNA qualitative analysis can be operated on spectrophotometer (Nanodrop Technologies). Since there is limited level and size of exosomal RNA compared to the complete cell, Agilent 2100 Bioanalyzer instrument is more recommended for higher accuracy and sensitivity to characterize RNA quality and concentration. The analysis process is operated on the chip and processed by software. After the complement of exosomal RNA quality and quantity estimation, RNA can be amplified to cDNA by QuantiTect Reverse Transcription kit (Qiagen) or SBI SeraMir Kit. Expression analysis of RNA in exosome of different sources can then be estimated by quantitative real-time (RT-qPCR), and microarray can be utilized as well. Moreover, next-generation sequencing can characterize whole transcriptome contained in exosomes, making it a powerful weapon for the current study of exosomal nucleic acids. Although blind as it may seem, this method can effectively help find novel significant sequence. The library preparation protocol mainly contains adapter ligation, cDNA synthesis, and PCR amplification. At the PCR amplification step, each RNA sequence is marked with a specific index primer and index (bar codes) which allows parallel sequencing in a flow cell along with other samples indexed with different sequences simultaneously. Amplified RNA libraries are then separated by run in a polyacrylamide gel electrophoresis. The amplified libraries can be analyzed on the Illumina sequencing platforms: HiSeq, MiSeq, and Genome Analyzer [110]. PCR-free efficient diagnosis methods are mostly probe-based, and mainly include microarray and molecular beacon. The microarray can recognize specific RNA sequence though the hybridization with more than 1000 Nucleic acid probe single distributed on microarray chip. Current RNA profiling chip mainly concludes Affymetrix Gene Chip miRNA Array 1.0 [189]. But this technology is not suitable for discovery of new RNA sequences and has an inferior transcript quantification ability compared to next-generation sequencing [189]. Molecular beacon (MB) is fluorescently labeled oligonucleotide chain with hairpin structure. Once the MB is bound with its complementary sequence, a strong fluorescence signal will be observed. It has been used in the detection of tubercle bacillus resistance genes as early as 20 years ago. It has also been used in the recent 5 years to identify mRNAs and microRNAs in exosome of lung cancer [190, 191], breast cancer [181, 192], pancreatic cancer [193], and prostate cancer. Only when beacons penetrate into exosome can they hybridize with targeted RNA. Making membrane permeabilization with streptolysin O (SLO) [191] or relying on MB’s own penetration [194] are both feasible.

Exosomal target miR-21 MB can directly penetrate into exosomes without need for saponin treatment [190]. Moreover the MB-based fluorescence detection technology has been able to accomplished simultaneous and multiple detection of miRNA inside the exosome from the serum of a high concentration (70% v/v) [190] or urine of 60% (v/v) [195], without need for exosome isolation or RNA extraction. The methodology of this technology is relatively mature, and the detail experiment process has been reported [194].

The DNA content in exosome is quite rare compared to RNA. Most methods in RNA analysis, like next-generation DNA sequencing, real time quantitative PCR, micro array etc. can be also used for DNA content detection in exosomes.

### Exosome lipid detection

Lipidology analysis techniques at cellular level have been developed maturely, and related review herein discusses different MS analyses in qualification and reproducibility aspects that have been published [196–198]. There are very few reports that concentrate on exosome lipid analyses methodology evaluation and innovation. This may be because of the relatively not rich biological function of exosome lipid. In the past decade, techniques including layer chromatography (TLC), gas liquid chromatography (GLC) and mass spectrometry (MS) have been mostly reported [199]. LC-MS based platform named micro LC Q-TOF MS has been demonstrated for urinary exosomes lipidology study [200]. High-throughput screening MS-based approach like ESI-MS (electrospray ionization-mass spectrometry) and MALDI-TOF (matrix-assisted laser desorption ionization-time of flight) have attracted more attention in the science community owing to their high efficiency and sensitivity for sample detection.

### Exosome glycan detection

There are more complex structures of macromolecules and relatively less various biological function of glycans, hence diverse and specific methods need to be developed. In brief, for general characterization of glycosylation, lectins are often employed at present. Lectins are proteins that bind to specific glycan structures. The lectins involved in glycosylation analysis technique contain blots [201], lectin arrays and lectin affinity purification.

## Conclusion and future perspective

Exosomes are small vesicles widely distributed in human body fluids. They are gradually and extensively accepted by the whole science community, in terms of their function in transferring biological molecules between cells, as well as their potential to become biomarkers for a series of diseases. Increasing studies have shown that exosomes play a key role in physiological or pathological processes, which also provides a theoretical basis for their use as a novel diagnostic tool. Various separation or detection methods are constantly being introduced at a booming speed. However, there is still a long way to go before exosomes become a routine testing item in tumor diagnosis.

### The establishment of standardized purification and detection method and discovery of exosome-associated tumor markers

The standard protocols for isolation and detection of exosomes are suitable for clinical applications, however, there are still major limitations to their clinical application. An ideal clinical method for detection of exosomes need to have the characteristics of high-throughput, short time-consumption, operability, high sensitivity, specificity, and results should be stable even at the interference of other biological substances, such as lipoprotein, apoptotic bodies and other extracellular vesicles. As summarized in Tables 1 and 2, there are many exosomal biomarkers that have come to light. However, owing to the lack of standard analysis method, many statistics are not comparable. Moreover, results from these small sample sized experiments are unconvincing when used to establish cut-off value or not to say evaluating diagnostic performance of every biomarker. Standardized research methods for exosomes should therefore be established as soon as possible, and novel biomarkers discovery should not be forgotten. At present, most protein biomarkers research is limited on membrane surface protein, while protein markers in exosome remain as a virgin land. Proteomics analysis will therefore contribute a lot in inner protein marker discovery.

### Single exosome detection is of great significance

Cells secrete more than one kind of exosome, which lead to high heterogeneity in exosomes [202, 203], and it’s well known that exosome compositions change with changing physiological state of parent cell. The detection of the whole exosome population cannot meet the needs for exploring the nature of the disease. Single exosome detection is always the future development direction. Meanwhile, numerous normal cells continuously release exosome, making it is very challenging to isolate and analyze the tumor-derived exosomes in such huge population. Most methods provide an average characteristic based on the whole exosome population detection, inducing information from tumor-derive exosome that may be submerged in signal pool, which is mainly consisted of the normal particles. It is not difficult to speculate that total exosome qualitative detection may never reach the goal of dynamic monitor of tumor progression as original intention of liquid biopsy. If one wants to apply exosome technology in clinical diagnosis as soon as possible, you must focus on the detection of tumor-derived exosomes subpopulation, and find more specific markers for tumor exosomes, by trying to eliminate interference from normal exosomes as much as possible. Optical tweezer technique may become a key for such problem, since it can trap only several exosomes in a light with certain wavelength. There are scientists [163] attempting to make measurement of exosome subpopulation via this method.

### Aptamer will play a more vital role in exosome detection

Exosomes can be purified before being tested to overcome the shortcomings of ordinary nucleic acid aptamers (without any modification) that are easily degraded or neutralized by related proteins in body fluids. And aptamer may own better prospect than antibody-based immune detection in realistic utilization, because: 1. Aptamers have both function of specific recognition and PCR/HCR (Hybridization Chain Reaction) based signal amplification. Nucleic acid amplification technology has rapidly developed, and the present used methods account for a small part in aptamer-based methods. 2. The weaker binding compared to antibodies makes aptamers very easy for exosome elution, with less impairment on exosome morphology and function. So, it is more conducive to use aptamers in researching on biological function of exosomes. 3. The aptamer targeted tumor exosome selective technique is similar to CELL-SELEX, and will help to find a new way for discovery of specific biomarkers except for complex MS. Moreover, the stability of heat and well-established synthesis, modifications and high-sensitivity analysis technologies, also make aptamers as perfect agents for exosome detection.

### Microfluidic technology is more suitable for the analysis of exosomes

The microfluidic method is the breaking point of exosomes testing in future clinical application. With low requirement for sample volume, the microfluidic method can achieve the goal of minimizing the size, cost, complexity of detection, accomplishing the whole reaction more quickly, and most of all, performing various experiments in a tiny space at the same time.

As mentioned above, growing number of researchers are moving ahead on this road, and there have been researchers who have designed microfluidic chips for immunocapture, by effectively combining the advantages of immunomagnetic beads and microfluidics chip. Even primitive as it may seem, it can stand for development orientation for future research, and above all, the bead-exosome complexes can be combined with characterization techniques, such as flow cytometry, electron microscopy, allowing qualitative detection during the process of isolation, and thus further saving examination time. Furthermore, how to connect multiple reactions seamlessly in a very small chip in a completely automatic manner remain to be a problem for follow-up researchers to think about. Lastly, the development of a perfect exosome detection instrument is inseparable from deep cooperation between engineers, clinicians, chemists and physicists. We believe that with continuous improvement of microfluidic technology, exosomes in clinical large-scale application will come to patient’s bed soon.

## Data Availability

Not applicable.
